# Supplementary material for: Comparative multiomics analysis of cell physiological state after culture in a basket bioreactor
Source: Sci Rep. 2022 Nov 23;12:20161. doi: 10.1038/s41598-022-24687-4 (PMC9686226; doi:10.1038/s41598-022-24687-4)
Supplement: Supplementary file 1 — Supplementary Information 1. [file 41598_2022_24687_MOESM1_ESM.zip › raw data/Transcriptomic raw data/6.Enrichment/2.KEGG/TE1vsTC1/UP/TE1vsTC1.up_KEGGenrich.html]

Pathway Enrichment


# The most enriched pathway terms

Statistic method: hypergeometric test

FDR correction method: Benjamini and Hochberg

| Term | Description | Sample number | Background number | P-value | Corrected P-value | Gene\_id | Gene\_name | KEGG\_ID |
| --- | --- | --- | --- | --- | --- | --- | --- | --- |
| csab04630 | JAK-STAT signaling pathway | 7/87 | 111/5900 | 0.00116844191602247 | 0.172885020619366 | 103226390 103235305 103246489 103236305 103237430 103247197 103238870 | IL6 IL11 CCND1 IL15 MYC MCL1 SOCS2 | csab:103226390 csab:103235305 csab:103246489 csab:103236305 csab:103237430 csab:103247197 csab:103238870 |
| csab00900 | Terpenoid backbone biosynthesis | 3/87 | 18/5900 | 0.00215334511436984 | 0.172885020619366 | 103240824 103215216 103223243 | ACAT2 HMGCS1 HMGCR | csab:103240824 csab:103215216 csab:103223243 |
| csab00100 | Steroid biosynthesis | 3/87 | 19/5900 | 0.00253002469199072 | 0.172885020619366 | 103237411 103226567 103215320 | SQLE CYP51A1 FDFT1 | csab:103237411 csab:103226567 csab:103215320 |
| csab00970 | Aminoacyl-tRNA biosynthesis | 4/87 | 43/5900 | 0.00351204542532956 | 0.17999232804814 | 103226274 103219751 103229694 103238568 | GARS1 IARS1 WARS1 MARS1 | csab:103226274 csab:103219751 csab:103229694 csab:103238568 |
| csab04012 | ErbB signaling pathway | 5/87 | 79/5900 | 0.00594876085104356 | 0.225827005522812 | 103235805 103244654 103235804 103237430 103228406 | AREG HBEGF EREG MYC CBLB | csab:103235805 csab:103244654 csab:103235804 csab:103237430 csab:103228406 |
| csab05210 | Colorectal cancer | 5/87 | 81/5900 | 0.0066095708933506 | 0.225827005522812 | 103235805 103224652 103246489 103235804 103237430 | AREG GADD45A CCND1 EREG MYC | csab:103235805 csab:103224652 csab:103246489 csab:103235804 csab:103237430 |
| csab04151 | PI3K-Akt signaling pathway | 10/87 | 280/5900 | 0.00775979158575501 | 0.227251039297111 | 103226390 103238335 103243277 103235805 103246489 103235804 103225530 103237430 103247197 103214795 | IL6 NR4A1 ITGB3 AREG CCND1 EREG EPHA2 MYC MCL1 COL4A1 | csab:103226390 csab:103238335 csab:103243277 csab:103235805 csab:103246489 csab:103235804 csab:103225530 csab:103237430 csab:103247197 csab:103214795 |
| csab05222 | Small cell lung cancer | 5/87 | 87/5900 | 0.00889924514675073 | 0.228043156885487 | 103224652 103246489 103237430 103214795 103242634 | GADD45A CCND1 MYC COL4A1 TRAF4 | csab:103224652 csab:103246489 csab:103237430 csab:103214795 csab:103242634 |
| csab05216 | Thyroid cancer | 3/87 | 32/5900 | 0.0112838540205839 | 0.257021119357744 | 103224652 103246489 103237430 | GADD45A CCND1 MYC | csab:103224652 csab:103246489 csab:103237430 |
| csab05219 | Bladder cancer | 3/87 | 38/5900 | 0.018016568386025 | 0.339527678470968 | 103244654 103246489 103237430 | HBEGF CCND1 MYC | csab:103244654 csab:103246489 csab:103237430 |
| csab05206 | MicroRNAs in cancer | 6/87 | 145/5900 | 0.0196508333925751 | 0.339527678470968 | 103214617 103243277 103246489 103237430 103247197 103214788 | SPRY2 ITGB3 CCND1 MYC MCL1 IRS2 | csab:103214617 csab:103243277 csab:103246489 csab:103237430 csab:103247197 csab:103214788 |
| csab00450 | Selenocompound metabolism | 2/87 | 15/5900 | 0.0199264986247099 | 0.339527678470968 | 103224624 103238568 | CTH MARS1 | csab:103224624 csab:103238568 |
| csab05410 | Hypertrophic cardiomyopathy | 4/87 | 74/5900 | 0.0232149958350469 | 0.339527678470968 | 103226390 103243277 103222156 103217090 | IL6 ITGB3 EDN1 CACNB4 | csab:103226390 csab:103243277 csab:103222156 csab:103217090 |
| csab00270 | Cysteine and methionine metabolism | 3/87 | 42/5900 | 0.0235096189932688 | 0.339527678470968 | 103218755 103224624 103224094 | BCAT1 CTH PHGDH | csab:103218755 csab:103224624 csab:103224094 |
| csab04350 | TGF-beta signaling pathway | 4/87 | 77/5900 | 0.0264163213017186 | 0.339527678470968 | 103246725 103237430 103237062 103245923 | BMP2 MYC E2F5 GREM1 | csab:103246725 csab:103237430 csab:103237062 csab:103245923 |
| csab00600 | Sphingolipid metabolism | 3/87 | 44/5900 | 0.0265598574994598 | 0.339527678470968 | 103236153 103230017 103219011 | UGT8 DEGS1 UGCG | csab:103236153 csab:103230017 csab:103219011 |
| csab04010 | MAPK signaling pathway | 8/87 | 247/5900 | 0.0284586495353785 | 0.339527678470968 | 103238335 103216504 103235805 103224652 103235804 103225530 103237430 103217090 | NR4A1 DUSP5 AREG GADD45A EREG EPHA2 MYC CACNB4 | csab:103238335 csab:103216504 csab:103235805 csab:103224652 csab:103235804 csab:103225530 csab:103237430 csab:103217090 |
| csab00280 | Valine, leucine and isoleucine degradation | 3/87 | 46/5900 | 0.0298121864023289 | 0.339527678470968 | 103218755 103240824 103215216 | BCAT1 ACAT2 HMGCS1 | csab:103218755 csab:103240824 csab:103215216 |
| csab04625 | C-type lectin receptor signaling pathway | 4/87 | 83/5900 | 0.0335819226841695 | 0.36233127106604 | 103226390 103215465 103216128 103228406 | IL6 EGR3 EGR2 CBLB | csab:103226390 csab:103215465 csab:103216128 csab:103228406 |
| csab00650 | Butanoate metabolism | 2/87 | 21/5900 | 0.0376477000790767 | 0.385888925810536 | 103240824 103215216 | ACAT2 HMGCS1 | csab:103240824 csab:103215216 |
| csab05213 | Endometrial cancer | 3/87 | 55/5900 | 0.0469117099755465 | 0.399919894343249 | 103224652 103246489 103237430 | GADD45A CCND1 MYC | csab:103224652 csab:103246489 csab:103237430 |
| csab04218 | Cellular senescence | 5/87 | 134/5900 | 0.047156883252648 | 0.399919894343249 | 103226390 103224652 103246489 103237430 103237062 | IL6 GADD45A CCND1 MYC E2F5 | csab:103226390 csab:103224652 csab:103246489 csab:103237430 csab:103237062 |
| csab00601 | Glycosphingolipid biosynthesis - lacto and neolacto series | 2/87 | 24/5900 | 0.0480985076613567 | 0.399919894343249 | 103234996 103217238 | FUT1 B3GALT1 | csab:103234996 csab:103217238 |
| csab04640 | Hematopoietic cell lineage | 3/87 | 56/5900 | 0.049055192404603 | 0.399919894343249 | 103226390 103235305 103243277 | IL6 IL11 ITGB3 | csab:103226390 csab:103235305 csab:103243277 |
| csab04933 | AGE-RAGE signaling pathway in diabetic complications | 4/87 | 95/5900 | 0.0510192083057898 | 0.399919894343249 | 103226390 103222156 103246489 103214795 | IL6 EDN1 CCND1 COL4A1 | csab:103226390 csab:103222156 csab:103246489 csab:103214795 |
| csab01230 | Biosynthesis of amino acids | 3/87 | 57/5900 | 0.0512461121586922 | 0.399919894343249 | 103218755 103224624 103224094 | BCAT1 CTH PHGDH | csab:103218755 csab:103224624 csab:103224094 |
| csab04668 | TNF signaling pathway | 4/87 | 98/5900 | 0.0560273214341684 | 0.399919894343249 | 103226390 103222156 103236305 103240296 | IL6 EDN1 IL15 TNFAIP3 | csab:103226390 csab:103222156 csab:103236305 csab:103240296 |
| csab04931 | Insulin resistance | 4/87 | 99/5900 | 0.0577539178726167 | 0.399919894343249 | 103226390 103247816 103214788 103220112 | IL6 TRIB3 IRS2 GFPT1 | csab:103226390 csab:103247816 csab:103214788 csab:103220112 |
| csab04390 | Hippo signaling pathway | 5/87 | 142/5900 | 0.057841950409131 | 0.399919894343249 | 103246725 103248148 103235805 103246489 103237430 | BMP2 DLG2 AREG CCND1 MYC | csab:103246725 csab:103248148 csab:103235805 csab:103246489 csab:103237430 |
| csab04115 | p53 signaling pathway | 3/87 | 61/5900 | 0.0604756913397108 | 0.399919894343249 | 103225266 103224652 103246489 | SESN2 GADD45A CCND1 | csab:103225266 csab:103224652 csab:103246489 |
| csab05323 | Rheumatoid arthritis | 3/87 | 61/5900 | 0.0604756913397108 | 0.399919894343249 | 103226390 103235305 103236305 | IL6 IL11 IL15 | csab:103226390 csab:103235305 csab:103236305 |
| csab05207 | Chemical carcinogenesis - receptor activation | 5/87 | 146/5900 | 0.0636679204841552 | 0.407872615601619 | 103219053 103246489 103214580 103237430 103218702 | KLF4 CCND1 KLF5 MYC MGST1 | csab:103219053 csab:103246489 csab:103214580 csab:103237430 csab:103218702 |
| csab05202 | Transcriptional misregulation in cancer | 5/87 | 148/5900 | 0.0667015429757315 | 0.414269299717915 | 103226390 103219133 103224652 103228427 103237430 | IL6 NR4A3 GADD45A NFKBIZ MYC | csab:103226390 csab:103219133 csab:103224652 csab:103228427 csab:103237430 |
| csab04919 | Thyroid hormone signaling pathway | 4/87 | 105/5900 | 0.0687080789776054 | 0.414269299717915 | 103243277 103246489 103237335 103237430 | ITGB3 CCND1 MED30 MYC | csab:103243277 csab:103246489 csab:103237335 csab:103237430 |
| csab04672 | Intestinal immune network for IgA production | 2/87 | 31/5900 | 0.0758845123513528 | 0.427557543113327 | 103226390 103236305 | IL6 IL15 | csab:103226390 csab:103236305 |
| csab05162 | Measles | 4/87 | 110/5900 | 0.0786020277300515 | 0.427557543113327 | 103226390 103246489 103240296 103228406 | IL6 CCND1 TNFAIP3 CBLB | csab:103226390 csab:103246489 csab:103240296 csab:103228406 |
| csab04110 | Cell cycle | 4/87 | 111/5900 | 0.0806626023399337 | 0.427557543113327 | 103224652 103246489 103237430 103237062 | GADD45A CCND1 MYC E2F5 | csab:103224652 csab:103246489 csab:103237430 csab:103237062 |
| csab04657 | IL-17 signaling pathway | 3/87 | 69/5900 | 0.0810710899804739 | 0.427557543113327 | 103226390 103240296 103242634 | IL6 TNFAIP3 TRAF4 | csab:103226390 csab:103240296 csab:103242634 |
| csab05169 | Epstein-Barr virus infection | 5/87 | 157/5900 | 0.0813402155191207 | 0.427557543113327 | 103226390 103224652 103246489 103237430 103240296 | IL6 GADD45A CCND1 MYC TNFAIP3 | csab:103226390 csab:103224652 csab:103246489 csab:103237430 csab:103240296 |
| csab04925 | Aldosterone synthesis and secretion | 3/87 | 70/5900 | 0.0838354498092679 | 0.429656680272498 | 103238335 103234364 103238835 | NR4A1 LDLR ATP2B1 | csab:103238335 csab:103234364 csab:103238835 |
| csab05220 | Chronic myeloid leukemia | 3/87 | 71/5900 | 0.0866401133405398 | 0.433200566702699 | 103224652 103246489 103237430 | GADD45A CCND1 MYC | csab:103224652 csab:103246489 csab:103237430 |
| csab04068 | FoxO signaling pathway | 4/87 | 117/5900 | 0.0935839178328032 | 0.447050253248819 | 103226390 103224652 103246489 103214788 | IL6 GADD45A CCND1 IRS2 | csab:103226390 csab:103224652 csab:103246489 csab:103214788 |
| csab05200 | Pathways in cancer | 10/87 | 428/5900 | 0.0973210892389635 | 0.447050253248819 | 103226390 103246725 103224652 103222156 103246489 103236305 103237430 103214795 103218702 103242634 | IL6 BMP2 GADD45A EDN1 CCND1 IL15 MYC COL4A1 MGST1 TRAF4 | csab:103226390 csab:103246725 csab:103224652 csab:103222156 csab:103246489 csab:103236305 csab:103237430 csab:103214795 csab:103218702 csab:103242634 |
| csab04913 | Ovarian steroidogenesis | 2/87 | 36/5900 | 0.0981329824204724 | 0.447050253248819 | 103234364 103237812 | LDLR LOC103237812 | csab:103234364 csab:103237812 |
| csab04979 | Cholesterol metabolism | 2/87 | 36/5900 | 0.0981329824204724 | 0.447050253248819 | 103234364 103229330 | LDLR NPC2 | csab:103234364 csab:103229330 |
| csab00260 | Glycine, serine and threonine metabolism | 2/87 | 37/5900 | 0.102782319602339 | 0.458051641706075 | 103224624 103224094 | CTH PHGDH | csab:103224624 csab:103224094 |
| csab04060 | Cytokine-cytokine receptor interaction | 5/87 | 170/5900 | 0.105269806586338 | 0.459155539365944 | 103226390 103235305 103246725 103236305 103221402 | IL6 IL11 BMP2 IL15 TNFRSF21 | csab:103226390 csab:103235305 csab:103246725 csab:103236305 csab:103221402 |
| csab05160 | Hepatitis C | 4/87 | 125/5900 | 0.112242866692301 | 0.47937057649837 | 103246489 103237430 103234364 103241901 | CCND1 MYC LDLR CLDN1 | csab:103246489 csab:103237430 csab:103234364 csab:103241901 |
| csab04930 | Type II diabetes mellitus | 2/87 | 41/5900 | 0.121951455935276 | 0.510205070749623 | 103238870 103214788 | SOCS2 IRS2 | csab:103238870 csab:103214788 |
| csab05166 | Human T-cell leukemia virus 1 infection | 5/87 | 183/5900 | 0.132310187699594 | 0.542471769568334 | 103226390 103246489 103236305 103237430 103216128 | IL6 CCND1 IL15 MYC EGR2 | csab:103226390 csab:103246489 csab:103236305 csab:103237430 csab:103216128 |
| csab00480 | Glutathione metabolism | 2/87 | 44/5900 | 0.136855989333533 | 0.550107408105378 | 103220822 103218702 | ODC1 MGST1 | csab:103220822 csab:103218702 |
| csab05161 | Hepatitis B | 4/87 | 138/5900 | 0.145757373748405 | 0.574620415738906 | 103226390 103215465 103237430 103216128 | IL6 EGR3 MYC EGR2 | csab:103226390 csab:103215465 csab:103237430 csab:103216128 |
| csab04310 | Wnt signaling pathway | 4/87 | 141/5900 | 0.154001398638994 | 0.595665787188563 | 103232583 103246489 103237430 103231576 | GPC4 CCND1 MYC TBL1X | csab:103232583 csab:103246489 csab:103237430 csab:103231576 |
| csab04927 | Cortisol synthesis and secretion | 2/87 | 50/5900 | 0.167743214491553 | 0.626650090170688 | 103238335 103234364 | NR4A1 LDLR | csab:103238335 csab:103234364 |
| csab05225 | Hepatocellular carcinoma | 4/87 | 146/5900 | 0.168125633948233 | 0.626650090170688 | 103224652 103246489 103237430 103218702 | GADD45A CCND1 MYC MGST1 | csab:103224652 csab:103246489 csab:103237430 csab:103218702 |
| csab04340 | Hedgehog signaling pathway | 2/87 | 52/5900 | 0.178294829620422 | 0.641235790740115 | 103219690 103246489 | GAS1 CCND1 | csab:103219690 csab:103246489 |
| csab04976 | Bile secretion | 2/87 | 52/5900 | 0.178294829620422 | 0.641235790740115 | 103234364 103223243 | LDLR HMGCR | csab:103234364 csab:103223243 |
| csab04970 | Salivary secretion | 2/87 | 53/5900 | 0.183609583813439 | 0.648964908306121 | 103238835 103244467 | ATP2B1 SLC12A2 | csab:103238835 csab:103244467 |
| csab01210 | 2-Oxocarboxylic acid metabolism | 1/87 | 14/5900 | 0.187962749282805 | 0.651219962855082 | 103218755 | BCAT1 | csab:103218755 |
| csab05217 | Basal cell carcinoma | 2/87 | 56/5900 | 0.199687369379866 | 0.651219962855082 | 103246725 103224652 | BMP2 GADD45A | csab:103246725 csab:103224652 |
| csab05221 | Acute myeloid leukemia | 2/87 | 57/5900 | 0.205085233278818 | 0.651219962855082 | 103246489 103237430 | CCND1 MYC | csab:103246489 csab:103237430 |
| csab04152 | AMPK signaling pathway | 3/87 | 107/5900 | 0.209059590385384 | 0.651219962855082 | 103246489 103223243 103214788 | CCND1 HMGCR IRS2 | csab:103246489 csab:103223243 csab:103214788 |
| csab04210 | Apoptosis | 3/87 | 107/5900 | 0.209059590385384 | 0.651219962855082 | 103224652 103247197 103235922 | GADD45A MCL1 PTPN13 | csab:103224652 csab:103247197 csab:103235922 |
| csab05218 | Melanoma | 2/87 | 58/5900 | 0.210499560920011 | 0.651219962855082 | 103224652 103246489 | GADD45A CCND1 | csab:103224652 csab:103246489 |
| csab00603 | Glycosphingolipid biosynthesis - globo and isoglobo series | 1/87 | 16/5900 | 0.211792529021719 | 0.651219962855082 | 103234996 | FUT1 | csab:103234996 |
| csab04917 | Prolactin signaling pathway | 2/87 | 60/5900 | 0.221371110826923 | 0.651219962855082 | 103246489 103238870 | CCND1 SOCS2 | csab:103246489 csab:103238870 |
| csab05230 | Central carbon metabolism in cancer | 2/87 | 60/5900 | 0.221371110826923 | 0.651219962855082 | 103237430 103233435 | MYC SLC7A5 | csab:103237430 csab:103233435 |
| csab00532 | Glycosaminoglycan biosynthesis - chondroitin sulfate / dermatan sulfate | 1/87 | 17/5900 | 0.223446854306408 | 0.651219962855082 | 103215414 | CSGALNACT1 | csab:103215414 |
| csab04910 | Insulin signaling pathway | 3/87 | 111/5900 | 0.224522599954865 | 0.651219962855082 | 103238870 103214788 103228406 | SOCS2 IRS2 CBLB | csab:103238870 csab:103214788 csab:103228406 |
| csab05418 | Fluid shear stress and atherosclerosis | 3/87 | 111/5900 | 0.224522599954865 | 0.651219962855082 | 103243277 103222156 103218702 | ITGB3 EDN1 MGST1 | csab:103243277 csab:103222156 csab:103218702 |
| csab05214 | Glioma | 2/87 | 62/5900 | 0.232289456311226 | 0.651219962855082 | 103224652 103246489 | GADD45A CCND1 | csab:103224652 csab:103246489 |
| csab05332 | Graft-versus-host disease | 1/87 | 18/5900 | 0.23493081209586 | 0.651219962855082 | 103226390 | IL6 | csab:103226390 |
| csab04972 | Pancreatic secretion | 2/87 | 63/5900 | 0.237762435153272 | 0.651219962855082 | 103238835 103244467 | ATP2B1 SLC12A2 | csab:103238835 csab:103244467 |
| csab05223 | Non-small cell lung cancer | 2/87 | 63/5900 | 0.237762435153272 | 0.651219962855082 | 103224652 103246489 | GADD45A CCND1 | csab:103224652 csab:103246489 |
| csab05205 | Proteoglycans in cancer | 4/87 | 169/5900 | 0.238251205922591 | 0.651219962855082 | 103243277 103244654 103246489 103237430 | ITGB3 HBEGF CCND1 MYC | csab:103243277 csab:103244654 csab:103246489 csab:103237430 |
| csab05163 | Human cytomegalovirus infection | 4/87 | 173/5900 | 0.251129319018076 | 0.674142783878471 | 103226390 103243277 103246489 103237430 | IL6 ITGB3 CCND1 MYC | csab:103226390 csab:103243277 csab:103246489 csab:103237430 |
| csab00770 | Pantothenate and CoA biosynthesis | 1/87 | 20/5900 | 0.257397437856295 | 0.674142783878471 | 103218755 | BCAT1 | csab:103218755 |
| csab04727 | GABAergic synapse | 2/87 | 67/5900 | 0.259713567414455 | 0.674142783878471 | 103238195 103238196 | SLC38A1 SLC38A2 | csab:103238195 csab:103238196 |
| csab05412 | Arrhythmogenic right ventricular cardiomyopathy | 2/87 | 68/5900 | 0.265209505126887 | 0.674142783878471 | 103243277 103217090 | ITGB3 CACNB4 | csab:103243277 csab:103217090 |
| csab05143 | African trypanosomiasis | 1/87 | 21/5900 | 0.268384924745156 | 0.674142783878471 | 103226390 | IL6 | csab:103226390 |
| csab03018 | RNA degradation | 2/87 | 69/5900 | 0.270706174235735 | 0.674142783878471 | 103242916 103234733 | TOB1 EXOSC5 | csab:103242916 csab:103234733 |
| csab00790 | Folate biosynthesis | 1/87 | 22/5900 | 0.279211682960358 | 0.674142783878471 | 103237812 | LOC103237812 | csab:103237812 |
| csab05224 | Breast cancer | 3/87 | 125/5900 | 0.280147781592364 | 0.674142783878471 | 103224652 103246489 103237430 | GADD45A CCND1 MYC | csab:103224652 csab:103246489 csab:103237430 |
| csab04510 | Focal adhesion | 4/87 | 182/5900 | 0.280623449597402 | 0.674142783878471 | 103243277 103246489 103214795 103224044 | ITGB3 CCND1 COL4A1 PIP5K1A | csab:103243277 csab:103246489 csab:103214795 csab:103224044 |
| csab04934 | Cushing syndrome | 3/87 | 127/5900 | 0.288224564626595 | 0.674142783878471 | 103238335 103246489 103234364 | NR4A1 CCND1 LDLR | csab:103238335 csab:103246489 csab:103234364 |
| csab05226 | Gastric cancer | 3/87 | 127/5900 | 0.288224564626595 | 0.674142783878471 | 103224652 103246489 103237430 | GADD45A CCND1 MYC | csab:103224652 csab:103246489 csab:103237430 |
| csab00630 | Glyoxylate and dicarboxylate metabolism | 1/87 | 23/5900 | 0.28988003675118 | 0.674142783878471 | 103240824 | ACAT2 | csab:103240824 |
| csab04975 | Fat digestion and absorption | 1/87 | 23/5900 | 0.28988003675118 | 0.674142783878471 | 103240824 | ACAT2 | csab:103240824 |
| csab05212 | Pancreatic cancer | 2/87 | 73/5900 | 0.2926766232448 | 0.674142783878471 | 103224652 103246489 | GADD45A CCND1 | csab:103224652 csab:103246489 |
| csab04974 | Protein digestion and absorption | 2/87 | 75/5900 | 0.303636569952395 | 0.676119541052382 | 103214795 103238196 | COL4A1 SLC38A2 | csab:103214795 csab:103238196 |
| csab05146 | Amoebiasis | 2/87 | 75/5900 | 0.303636569952395 | 0.676119541052382 | 103226390 103214795 | IL6 COL4A1 | csab:103226390 csab:103214795 |
| csab04211 | Longevity regulating pathway | 2/87 | 76/5900 | 0.309106563971964 | 0.676119541052382 | 103225266 103214788 | SESN2 IRS2 | csab:103225266 csab:103214788 |
| csab05414 | Dilated cardiomyopathy | 2/87 | 76/5900 | 0.309106563971964 | 0.676119541052382 | 103243277 103217090 | ITGB3 CACNB4 | csab:103243277 csab:103217090 |
| csab01523 | Antifolate resistance | 1/87 | 25/5900 | 0.310750662423494 | 0.676119541052382 | 103226390 | IL6 | csab:103226390 |
| csab04666 | Fc gamma R-mediated phagocytosis | 2/87 | 77/5900 | 0.314568593439803 | 0.676119541052382 | 103240591 103224044 | WASF1 PIP5K1A | csab:103240591 csab:103224044 |
| csab04530 | Tight junction | 3/87 | 134/5900 | 0.316621833858677 | 0.676119541052382 | 103248148 103246489 103241901 | DLG2 CCND1 CLDN1 | csab:103248148 csab:103246489 csab:103241901 |
| csab04064 | NF-kappa B signaling pathway | 2/87 | 79/5900 | 0.325465001556033 | 0.687838405350379 | 103224652 103240296 | GADD45A TNFAIP3 | csab:103224652 csab:103240296 |
| csab04512 | ECM-receptor interaction | 2/87 | 81/5900 | 0.336318566100449 | 0.698895816883893 | 103243277 103214795 | ITGB3 COL4A1 | csab:103243277 csab:103214795 |
| csab00250 | Alanine, aspartate and glutamate metabolism | 1/87 | 28/5900 | 0.34092478872385 | 0.698895816883893 | 103220112 | GFPT1 | csab:103220112 |
| csab04710 | Circadian rhythm | 1/87 | 28/5900 | 0.34092478872385 | 0.698895816883893 | 103227939 | BHLHE40 | csab:103227939 |
| csab04066 | HIF-1 signaling pathway | 2/87 | 83/5900 | 0.347122502786621 | 0.704555574962944 | 103226390 103222156 | IL6 EDN1 | csab:103226390 csab:103222156 |
| csab05231 | Choline metabolism in cancer | 2/87 | 85/5900 | 0.357870451959991 | 0.719249437762727 | 103240591 103224044 | WASF1 PIP5K1A | csab:103240591 csab:103224044 |
| csab01522 | Endocrine resistance | 2/87 | 87/5900 | 0.368556460043143 | 0.723570295065171 | 103244654 103246489 | HBEGF CCND1 | csab:103244654 csab:103246489 |
| csab04724 | Glutamatergic synapse | 2/87 | 87/5900 | 0.368556460043143 | 0.723570295065171 | 103238195 103238196 | SLC38A1 SLC38A2 | csab:103238195 csab:103238196 |
| csab05167 | Kaposi sarcoma-associated herpesvirus infection | 3/87 | 148/5900 | 0.373483468438852 | 0.723570295065171 | 103226390 103246489 103237430 | IL6 CCND1 MYC | csab:103226390 csab:103246489 csab:103237430 |
| csab01200 | Carbon metabolism | 2/87 | 89/5900 | 0.379174961683373 | 0.723570295065171 | 103224094 103240824 | PHGDH ACAT2 | csab:103224094 csab:103240824 |
| csab05203 | Viral carcinogenesis | 3/87 | 155/5900 | 0.401656542417343 | 0.723570295065171 | 103215465 103246489 103216128 | EGR3 CCND1 EGR2 | csab:103215465 csab:103246489 csab:103216128 |
| csab00620 | Pyruvate metabolism | 1/87 | 35/5900 | 0.406346794825597 | 0.723570295065171 | 103240824 | ACAT2 | csab:103240824 |
| csab00830 | Retinol metabolism | 1/87 | 35/5900 | 0.406346794825597 | 0.723570295065171 | 103231285 | ALDH1A3 | csab:103231285 |
| csab01250 | Biosynthesis of nucleotide sugars | 1/87 | 35/5900 | 0.406346794825597 | 0.723570295065171 | 103220112 | GFPT1 | csab:103220112 |
| csab05144 | Malaria | 1/87 | 35/5900 | 0.406346794825597 | 0.723570295065171 | 103226390 | IL6 | csab:103226390 |
| csab00565 | Ether lipid metabolism | 1/87 | 36/5900 | 0.415152903751458 | 0.723570295065171 | 103236153 | UGT8 | csab:103236153 |
| csab00982 | Drug metabolism - cytochrome P450 | 1/87 | 36/5900 | 0.415152903751458 | 0.723570295065171 | 103218702 | MGST1 | csab:103218702 |
| csab04978 | Mineral absorption | 1/87 | 36/5900 | 0.415152903751458 | 0.723570295065171 | 103238835 | ATP2B1 | csab:103238835 |
| csab00071 | Fatty acid degradation | 1/87 | 37/5900 | 0.423829864422267 | 0.723570295065171 | 103240824 | ACAT2 | csab:103240824 |
| csab04270 | Vascular smooth muscle contraction | 2/87 | 99/5900 | 0.431085151751005 | 0.723570295065171 | 103222156 103239748 | EDN1 CALCB | csab:103222156 csab:103239748 |
| csab00330 | Arginine and proline metabolism | 1/87 | 38/5900 | 0.432379549190348 | 0.723570295065171 | 103220822 | ODC1 | csab:103220822 |
| csab00380 | Tryptophan metabolism | 1/87 | 38/5900 | 0.432379549190348 | 0.723570295065171 | 103240824 | ACAT2 | csab:103240824 |
| csab00514 | Other types of O-glycan biosynthesis | 1/87 | 38/5900 | 0.432379549190348 | 0.723570295065171 | 103227904 | GXYLT2 | csab:103227904 |
| csab04961 | Endocrine and other factor-regulated calcium reabsorption | 1/87 | 38/5900 | 0.432379549190348 | 0.723570295065171 | 103238835 | ATP2B1 | csab:103238835 |
| csab05204 | Chemical carcinogenesis - DNA adducts | 1/87 | 38/5900 | 0.432379549190348 | 0.723570295065171 | 103218702 | MGST1 | csab:103218702 |
| csab05171 | Coronavirus disease - COVID-19 | 3/87 | 163/5900 | 0.433430909705705 | 0.723570295065171 | 103226390 103244654 103221259 | IL6 HBEGF RPL22L1 | csab:103226390 csab:103244654 csab:103221259 |
| csab00140 | Steroid hormone biosynthesis | 1/87 | 39/5900 | 0.440803803578005 | 0.723570295065171 | 103237812 | LOC103237812 | csab:103237812 |
| csab04623 | Cytosolic DNA-sensing pathway | 1/87 | 39/5900 | 0.440803803578005 | 0.723570295065171 | 103226390 | IL6 | csab:103226390 |
| csab04936 | Alcoholic liver disease | 2/87 | 101/5900 | 0.441201399429982 | 0.723570295065171 | 103226390 103246489 | IL6 CCND1 | csab:103226390 csab:103246489 |
| csab04935 | Growth hormone synthesis, secretion and action | 2/87 | 102/5900 | 0.44622335974509 | 0.724932374525828 | 103238870 103214788 | SOCS2 IRS2 | csab:103238870 csab:103214788 |
| csab05416 | Viral myocarditis | 1/87 | 40/5900 | 0.449104446657464 | 0.724932374525828 | 103246489 | CCND1 | csab:103246489 |
| csab04514 | Cell adhesion molecules | 2/87 | 106/5900 | 0.466061284567483 | 0.746330111684271 | 103220287 103241901 | NRXN1 CLDN1 | csab:103220287 csab:103241901 |
| csab00590 | Arachidonic acid metabolism | 1/87 | 43/5900 | 0.473282509848562 | 0.746330111684271 | 103237812 | LOC103237812 | csab:103237812 |
| csab05321 | Inflammatory bowel disease | 1/87 | 43/5900 | 0.473282509848562 | 0.746330111684271 | 103226390 | IL6 | csab:103226390 |
| csab00520 | Amino sugar and nucleotide sugar metabolism | 1/87 | 44/5900 | 0.481106382418679 | 0.747172790877495 | 103220112 | GFPT1 | csab:103220112 |
| csab05134 | Legionellosis | 1/87 | 44/5900 | 0.481106382418679 | 0.747172790877495 | 103226390 | IL6 | csab:103226390 |
| csab00980 | Metabolism of xenobiotics by cytochrome P450 | 1/87 | 45/5900 | 0.488815355220861 | 0.748266726060374 | 103218702 | MGST1 | csab:103218702 |
| csab04926 | Relaxin signaling pathway | 2/87 | 111/5900 | 0.490271532408137 | 0.748266726060374 | 103222156 103214795 | EDN1 COL4A1 | csab:103222156 csab:103214795 |
| csab05135 | Yersinia infection | 2/87 | 112/5900 | 0.495032385784332 | 0.748266726060374 | 103226390 103224044 | IL6 PIP5K1A | csab:103226390 csab:103224044 |
| csab00983 | Drug metabolism - other enzymes | 1/87 | 46/5900 | 0.496411096313224 | 0.748266726060374 | 103218702 | MGST1 | csab:103218702 |
| csab04810 | Regulation of actin cytoskeleton | 3/87 | 181/5900 | 0.502511265278112 | 0.751932915197175 | 103243277 103240591 103224044 | ITGB3 WASF1 PIP5K1A | csab:103243277 csab:103240591 csab:103224044 |
| csab04261 | Adrenergic signaling in cardiomyocytes | 2/87 | 115/5900 | 0.509148121460921 | 0.75195922199649 | 103217090 103238835 | CACNB4 ATP2B1 | csab:103217090 csab:103238835 |
| csab04923 | Regulation of lipolysis in adipocytes | 1/87 | 48/5900 | 0.511269436264193 | 0.75195922199649 | 103214788 | IRS2 | csab:103214788 |
| csab04550 | Signaling pathways regulating pluripotency of stem cells | 2/87 | 116/5900 | 0.513797044447268 | 0.75195922199649 | 103219053 103237430 | KLF4 MYC | csab:103219053 csab:103237430 |
| csab00240 | Pyrimidine metabolism | 1/87 | 49/5900 | 0.518535252915768 | 0.75195922199649 | 103225017 | CTPS1 | csab:103225017 |
| csab01212 | Fatty acid metabolism | 1/87 | 50/5900 | 0.525694274108099 | 0.75195922199649 | 103240824 | ACAT2 | csab:103240824 |
| csab04061 | Viral protein interaction with cytokine and cytokine receptor | 1/87 | 50/5900 | 0.525694274108099 | 0.75195922199649 | 103226390 | IL6 | csab:103226390 |
| csab04924 | Renin secretion | 1/87 | 51/5900 | 0.532748051570081 | 0.75195922199649 | 103222156 | EDN1 | csab:103222156 |
| csab01240 | Biosynthesis of cofactors | 2/87 | 121/5900 | 0.536610937410793 | 0.75195922199649 | 103218755 103225017 | BCAT1 CTPS1 | csab:103218755 csab:103225017 |
| csab04921 | Oxytocin signaling pathway | 2/87 | 122/5900 | 0.541086476027283 | 0.75195922199649 | 103246489 103217090 | CCND1 CACNB4 | csab:103246489 csab:103217090 |
| csab03320 | PPAR signaling pathway | 1/87 | 53/5900 | 0.546545971109644 | 0.75195922199649 | 103215216 | HMGCS1 | csab:103215216 |
| csab04213 | Longevity regulating pathway - multiple species | 1/87 | 53/5900 | 0.546545971109644 | 0.75195922199649 | 103214788 | IRS2 | csab:103214788 |
| csab05133 | Pertussis | 1/87 | 53/5900 | 0.546545971109644 | 0.75195922199649 | 103226390 | IL6 | csab:103226390 |
| csab04022 | cGMP-PKG signaling pathway | 2/87 | 127/5900 | 0.56302107421007 | 0.767671038625988 | 103238835 103214788 | ATP2B1 IRS2 | csab:103238835 csab:103214788 |
| csab05132 | Salmonella infection | 3/87 | 199/5900 | 0.56718641589871 | 0.767671038625988 | 103226390 103237430 103220717 | IL6 MYC RHOB | csab:103226390 csab:103237430 csab:103220717 |
| csab00310 | Lysine degradation | 1/87 | 57/5900 | 0.572944726389152 | 0.767671038625988 | 103240824 | ACAT2 | csab:103240824 |
| csab04920 | Adipocytokine signaling pathway | 1/87 | 57/5900 | 0.572944726389152 | 0.767671038625988 | 103214788 | IRS2 | csab:103214788 |
| csab04621 | NOD-like receptor signaling pathway | 2/87 | 131/5900 | 0.580031665064201 | 0.76771077040432 | 103226390 103240296 | IL6 TNFAIP3 | csab:103226390 csab:103240296 |
| csab04150 | mTOR signaling pathway | 2/87 | 132/5900 | 0.584209171624751 | 0.76771077040432 | 103225266 103233435 | SESN2 SLC7A5 | csab:103225266 csab:103233435 |
| csab04932 | Non-alcoholic fatty liver disease | 2/87 | 132/5900 | 0.584209171624751 | 0.76771077040432 | 103226390 103214788 | IL6 IRS2 | csab:103226390 csab:103214788 |
| csab01524 | Platinum drug resistance | 1/87 | 61/5900 | 0.597823267127333 | 0.774733481952383 | 103218702 | MGST1 | csab:103218702 |
| csab04260 | Cardiac muscle contraction | 1/87 | 61/5900 | 0.597823267127333 | 0.774733481952383 | 103217090 | CACNB4 | csab:103217090 |
| csab05165 | Human papillomavirus infection | 4/87 | 281/5900 | 0.600890846977702 | 0.774733481952383 | 103243277 103248148 103246489 103214795 | ITGB3 DLG2 CCND1 COL4A1 | csab:103243277 csab:103248148 csab:103246489 csab:103214795 |
| csab04144 | Endocytosis | 3/87 | 214/5900 | 0.617079853483712 | 0.790633562276006 | 103234364 103224044 103228406 | LDLR PIP5K1A CBLB | csab:103234364 csab:103224044 csab:103228406 |
| csab05100 | Bacterial invasion of epithelial cells | 1/87 | 66/5900 | 0.626915078809255 | 0.798245907800604 | 103240591 | WASF1 | csab:103240591 |
| csab04520 | Adherens junction | 1/87 | 67/5900 | 0.632478738072812 | 0.800358896943991 | 103240591 | WASF1 | csab:103240591 |
| csab01521 | EGFR tyrosine kinase inhibitor resistance | 1/87 | 69/5900 | 0.643361165388517 | 0.80593862424792 | 103226390 | IL6 | csab:103226390 |
| csab00562 | Inositol phosphate metabolism | 1/87 | 70/5900 | 0.648682307321496 | 0.80593862424792 | 103224044 | PIP5K1A | csab:103224044 |
| csab01232 | Nucleotide metabolism | 1/87 | 70/5900 | 0.648682307321496 | 0.80593862424792 | 103225017 | CTPS1 | csab:103225017 |
| csab04912 | GnRH signaling pathway | 1/87 | 73/5900 | 0.664179332951406 | 0.820221465391796 | 103244654 | HBEGF | csab:103244654 |
| csab04620 | Toll-like receptor signaling pathway | 1/87 | 75/5900 | 0.674133257905805 | 0.82752884952509 | 103226390 | IL6 | csab:103226390 |
| csab04916 | Melanogenesis | 1/87 | 76/5900 | 0.67900028049159 | 0.82854200893319 | 103222156 | EDN1 | csab:103222156 |
| csab05417 | Lipid and atherosclerosis | 2/87 | 160/5900 | 0.688985527310189 | 0.829949490671309 | 103226390 103234364 | IL6 LDLR | csab:103226390 csab:103234364 |
| csab04024 | cAMP signaling pathway | 2/87 | 161/5900 | 0.692299331242897 | 0.829949490671309 | 103222156 103238835 | EDN1 ATP2B1 | csab:103222156 csab:103238835 |
| csab04360 | Axon guidance | 2/87 | 161/5900 | 0.692299331242897 | 0.829949490671309 | 103225530 103246295 | EPHA2 SLIT2 | csab:103225530 csab:103246295 |
| csab04660 | T cell receptor signaling pathway | 1/87 | 80/5900 | 0.6977600981772 | 0.831632675153058 | 103228406 | CBLB | csab:103228406 |
| csab04659 | Th17 cell differentiation | 1/87 | 81/5900 | 0.702278117328159 | 0.832179271978454 | 103226390 | IL6 | csab:103226390 |
| csab04070 | Phosphatidylinositol signaling system | 1/87 | 83/5900 | 0.711114813424191 | 0.837807682482524 | 103224044 | PIP5K1A | csab:103224044 |
| csab05215 | Prostate cancer | 1/87 | 85/5900 | 0.719692153251965 | 0.841872428106459 | 103246489 | CCND1 | csab:103246489 |
| csab05142 | Chagas disease | 1/87 | 86/5900 | 0.723885924991789 | 0.841872428106459 | 103226390 | IL6 | csab:103226390 |
| csab04015 | Rap1 signaling pathway | 2/87 | 172/5900 | 0.726884974511431 | 0.841872428106459 | 103243277 103225530 | ITGB3 EPHA2 | csab:103243277 csab:103225530 |
| csab04020 | Calcium signaling pathway | 2/87 | 176/5900 | 0.738633887431246 | 0.847542929220908 | 103215153 103238835 | GDNF ATP2B1 | csab:103215153 csab:103238835 |
| csab05145 | Toxoplasmosis | 1/87 | 90/5900 | 0.740049679661183 | 0.847542929220908 | 103234364 | LDLR | csab:103234364 |
| csab04928 | Parathyroid hormone synthesis, secretion and action | 1/87 | 93/5900 | 0.751555270802255 | 0.855937947302568 | 103244654 | HBEGF | csab:103244654 |
| csab03010 | Ribosome | 1/87 | 95/5900 | 0.758944494219221 | 0.859578018314588 | 103221259 | RPL22L1 | csab:103221259 |
| csab05208 | Chemical carcinogenesis - reactive oxygen species | 2/87 | 188/5900 | 0.771355202075307 | 0.859866025414564 | 103218702 103237812 | MGST1 LOC103237812 | csab:103218702 csab:103237812 |
| csab04145 | Phagosome | 1/87 | 100/5900 | 0.776480559771993 | 0.859866025414564 | 103243277 | ITGB3 | csab:103243277 |
| csab04380 | Osteoclast differentiation | 1/87 | 101/5900 | 0.779833351375413 | 0.859866025414564 | 103243277 | ITGB3 | csab:103243277 |
| csab04670 | Leukocyte transendothelial migration | 1/87 | 101/5900 | 0.779833351375413 | 0.859866025414564 | 103241901 | CLDN1 | csab:103241901 |
| csab04071 | Sphingolipid signaling pathway | 1/87 | 102/5900 | 0.783136420599476 | 0.859866025414564 | 103230017 | DEGS1 | csab:103230017 |
| csab04915 | Estrogen signaling pathway | 1/87 | 103/5900 | 0.786390496385582 | 0.859866025414564 | 103244654 | HBEGF | csab:103244654 |
| csab04217 | Necroptosis | 1/87 | 105/5900 | 0.792754530748062 | 0.859866025414564 | 103240296 | TNFAIP3 | csab:103240296 |
| csab04611 | Platelet activation | 1/87 | 105/5900 | 0.792754530748062 | 0.859866025414564 | 103243277 | ITGB3 | csab:103243277 |
| csab04371 | Apelin signaling pathway | 1/87 | 113/5900 | 0.81638962109422 | 0.879543179253557 | 103246489 | CCND1 | csab:103246489 |
| csab04080 | Neuroactive ligand-receptor interaction | 2/87 | 210/5900 | 0.822252380717463 | 0.879543179253557 | 103222156 103239748 | EDN1 CALCB | csab:103222156 csab:103239748 |
| csab05164 | Influenza A | 1/87 | 116/5900 | 0.824548167147595 | 0.879543179253557 | 103226390 | IL6 | csab:103226390 |
| csab04613 | Neutrophil extracellular trap formation | 1/87 | 118/5900 | 0.829787036565364 | 0.879543179253557 | 103243277 | ITGB3 | csab:103243277 |
| csab04142 | Lysosome | 1/87 | 119/5900 | 0.832348179391171 | 0.879543179253557 | 103229330 | NPC2 | csab:103229330 |
| csab04140 | Autophagy - animal | 1/87 | 126/5900 | 0.849240181793077 | 0.888463219732805 | 103214788 | IRS2 | csab:103214788 |
| csab04072 | Phospholipase D signaling pathway | 1/87 | 128/5900 | 0.853749502650299 | 0.888463219732805 | 103224044 | PIP5K1A | csab:103224044 |
| csab05152 | Tuberculosis | 1/87 | 129/5900 | 0.855953902038626 | 0.888463219732805 | 103226390 | IL6 | csab:103226390 |
| csab04120 | Ubiquitin mediated proteolysis | 1/87 | 130/5900 | 0.858125451254124 | 0.888463219732805 | 103228406 | CBLB | csab:103228406 |
| csab04141 | Protein processing in endoplasmic reticulum | 1/87 | 147/5900 | 0.890458467264178 | 0.917306461252043 | 103233048 | HERPUD1 | csab:103233048 |
| csab05415 | Diabetic cardiomyopathy | 1/87 | 169/5900 | 0.921707764198654 | 0.94475045830362 | 103220112 | GFPT1 | csab:103220112 |
| csab04014 | Ras signaling pathway | 1/87 | 184/5900 | 0.937777882919122 | 0.956364479861965 | 103225530 | EPHA2 | csab:103225530 |
| csab05010 | Alzheimer disease | 2/87 | 302/5900 | 0.942368902107887 | 0.956364479861965 | 103226390 103214788 | IL6 IRS2 | csab:103226390 csab:103214788 |
| csab05020 | Prion disease | 1/87 | 211/5900 | 0.958914830243461 | 0.968362266994628 | 103226390 | IL6 | csab:103226390 |
| csab05168 | Herpes simplex virus 1 infection | 2/87 | 410/5900 | 0.986236003238834 | 0.991070493450789 | 103226390 103243277 | IL6 ITGB3 | csab:103226390 csab:103243277 |
| csab05022 | Pathways of neurodegeneration - multiple diseases | 1/87 | 373/5900 | 0.996736493609993 | 0.996736493609993 | 103226390 | IL6 | csab:103226390 |
